# Risk of incident active tuberculosis disease in patients treated with non-steroidal anti-inflammatory drugs: a population-based study

**DOI:** 10.1186/s12890-017-0425-3

**Published:** 2017-05-04

**Authors:** Chun-Wei Wu, Jiunn-Yih Wu, Meng-Tse Gabriel Lee, Chih-Cheng Lai, I-Lin Wu, Yi-Wen Tsai, Shy-Shin Chang, Chien-Chang Lee

**Affiliations:** 1 Department of Emergency Medicine, Chang Gung Memorial Hospital, Keelung, Taiwan and Chang Gung University College of Medicine, Taoyuan, Taiwan; 2 Department of Emergency Medicine, National Taiwan University Hospital, Number 7, Chung-Shan South Road, Taipei, 100 Taiwan; 3 Department of Intensive Care Medicine, Chi Mei Medical Center, Liouying, Tainan, Taiwan; 4 Department of Emergency Medicine, Taoyuan Armed Forces General Hospital, Taoyuan, Taiwan; 5 Department of Family Medicine, Chang Gung Memorial Hospital, Keelung and Chang Gung University College of Medicine, Taoyuan, Taiwan; 6 Department of Family Medicine, Taipei Medical University Hospital and School of Medicine, Taipei Medical University, Taipei, 110 Taiwan; 7 Department of Family Medicine, Taipei Medical University Hospital, No.252, Wuxing St, Xinyi District, Taipei, Taiwan

**Keywords:** Tuberculosis, NSAIDs, Coxibs

## Abstract

**Background:**

*Mycobacterium tuberculosis* (TB) is one of the world’s most devastating public health threats. Our goal is to evaluate whether the use of non-steroidal anti-inflammatory drugs (NSAIDs) affect the risk of new incident active TB disease.

**Methods:**

We conducted a nested case-control analysis by using a 1 million longitudinally followed cohort, from Taiwan’s national health insurance research database. Effects of NSAIDs on active TB were estimated by conditional logistic regression and adjusted using a TB-specific disease risk score (DRS). NSAIDs exposures were defined as having a prescription record of NSAIDs ≧ 7 days that ended between 31 and 90 days prior to the index date.

**Results:**

A total of 123,419 users of traditional NSAIDs, 16,392 users of cyclooxygenase-2 selective inhibitor (Coxibs), and 4706 incident cases of active TB were identified. Compared with nonusers, use of traditional NSAIDs was associated with an increased risk of TB in the unadjusted analysis ([RR], 1.39; 95% [CI], 1.24 – 1.57 and DRS adjusted analysis ([ARR], 1.30; 95% [CI], 1.15– 1.47). However, use of Coxibs was not associated with a significant increase in the risk of TB after DRS adjustment ([ARR], 1.23; 95% [CI], 0.89 – 1.70).

**Conclusions:**

In this large population-based study, we found that subjects using traditional NSAIDs were associated with increased risk for active TB. We did not find evidence for a causative mechanism between traditional NSAIDs and TB, and more research is required to verify whether the association between traditional NSAIDs and TB is causal, or simply reflects an increased use of anti-inflammatory drugs in the early phases of TB onset.

**Electronic supplementary material:**

The online version of this article (doi:10.1186/s12890-017-0425-3) contains supplementary material, which is available to authorized users.

## Background

*Mycobacterium tuberculosis* (TB), the causative bacterium pathogen, is one of the world’s most devastating public health threats. In 2013, there are approximately 9 million cases of new active TB, and an associated death of 1.5 million [1, 2]. It is WHO’s Global Plan to eradicate TB by the year of 2050, but it is difficult to achieve that goal by the current rate of decrease in TB infection. Our goal is to find out whether Non-steroidal anti-inflammatory drugs (NSAIDs), which is one of the most commonly used drugs in the world, might affect the risk of active TB [3–5]. Use of traditional NSAIDs is considered safe and they can be bought over the counter to relieve pain and fever. The therapeutic effects of traditional NSAIDs are primarily attained through the inhibition of the cyclooxygenase-1 (COX-1) and cyclooxygenase-2 (COX-2) enzymes, which are critical mediators of pain, inflammation, and fever [6–8]. Specific COX-2 inhibitors (Coxibs) are the newer generation of NSAIDs that selectively inhibit the COX-2 enzyme. Use of Coxibs is associated with less gastrointestinal complication than traditional NSAIDs and their use often requires a prescription [9–11].

As far as we were aware of, there is only one small-scale observation study that investigates the direct relationship between use of NSAIDs and active TB onset, and two small-scale observation studies that we can infer the relationship between NSAIDs and active TB onset [12–14]. In 1984, the Bass group showed that in 38 latent TB patients, the chance of developing active TB is directly increased with the use of NSAIDs [13]. Unfortunately, no risk estimate was provided due to the small sample size. In the 2009 Brassard paper, the group reported that NSAID is the most frequently used nonbiologic anti- rheumatoid arthritis medication for the 50 rheumatoid arthritis patients that developed TB. In addition, use of NSAID is associated with 1.2 fold increase in the risk of active TB [14]. In a more recent study by Chang et al., the group reported that NSAID is the most frequently used traditional systemic antipsoriatic drugs for the 497 psoriasis patients that developed TB, and frequent users of traditional NSAIDs were found to have 1.85 fold increase in TB risk [12]. Just like the bass study, the Chang study has a limited sample size and only looked at patients with psoriasis. Hence, we wish to investigate the influence of NSAIDs on active TB in the general population.

Taiwan has one of the most affordable single-payer universal public health insurance and the claim history is completely recorded in a public database. Since the cost of a physician prescribed NSAID (about $4-8US) is often similar to the self acquisition cost of NSAID over the counter, we believe that there are minimal over the counter use of traditional NSAIDs [15]. As a result, the Taiwanese National Health Insurance Research Database (NHIRD) of Taiwan, a national representative longitudinal cohort, is a great data source for analyzing the risk of new incident active tuberculosis disease in patients treated with NSAIDs or Coxibs. We, therefore, carried out a population-based study using the 1 million national cohort samples.

## Methods

### Data source

We carried out a population-based study using the National Health Insurance Research Database (NHIRD) of Taiwan, done under the approval of the institutional review board of National Taiwan University Hospital. The NHIRD database contains de-identified secondary data, and met the requirements of the “Personal Information Protection Act” in Taiwan. Thus, the data were analyzed anonymously and the need for informed consent was waived. NHIRD records the complete claim history of 1 million randomly selected participants enrolled in Taiwan’s National Health Insurance (NHI), which is a single-payer universal public health insurance [15]. These 1 million participants are believed to be representative of the entire Taiwanese population.

Several studies showed that the NHIRD database is appropriate for use in pharmacoepidemiologic research [12, 16, 17]. The NHIRD database contains participant demographics and detail claim history, which includes medications prescribed, medications quantity, route of administration, individual diagnoses, operations, and outpatient and inpatient electronic claim records.

### Study cohort

We used a study cohort that consists of a longitudinally followed Taiwanese’ population from January 1999 to December 2011. A new-user cohort design was adopted by excluding existing users of NSAID and prevalent cases of TB in the year 1999 (pre-enrollment period) [18]. Patients entered the cohort on the first day of the year 2000 and were followed up until the first occurrence of any of these events: diagnosis of active TB, termination of health insurance coverage, death, or end of the study period. People who were less than 18 years old on January 1, 2000, were not eligible for entering the cohort until they became 18 years old in later years. This cohort design thus allows a clean selection of case and controls, in which all patients investigated are new users of NSAIDs and have new cases of active TB.

### Selection of cases and controls

Patients with new onset active TB disease were identified using the following criteria: at least one outpatient visit or one hospital admission with ICD-9-CM codes of TB (010-018, including all subcategories), plus the prescription of more than two anti-tuberculosis medications for more than 28 days. We excluded patients with a subsequent diagnosis of nontuberculosis mycobacterial infection or lung cancer. This is because the diagnosis of nontuberculosis lung cancer is clinically difficult, and lung cancer has been associated with an increase in the risk of TB. Our case definition had been used in previous studies and had been validated in a linked survey database [19, 20]. One hundred controls were selected to match cases on index date, 5-year age group, and gender using risk set sampling scheme. Index date referred to the first date of TB diagnosis. The one-year period preceding index date was used for assessment of NSAID exposure status.

### Medication exposure

NSAIDs exposures were defined as having a prescription record of NSAID ≧ 7 days. By restricting to NSAID users having a drug prescription record ≧ 7 days, we found that most of the NSAID users were associated with codes for arthritis and other rheumatic conditions. Few patients were associated with codes for fever. As NSAID may be used to treat the early symptoms of active TB, we defined use of NSAID as a prescription that ended between 31 and 90 days prior to TB diagnosis to avoid the possible reverse causation between use of NSAID and active TB. Traditional NSAIDs are defined as drugs with any of the following compounds: ibuprofen, naproxen, ketoprofen, dexibuprofen, piroxicam, tolfenamic acid, diclofenac, etodolac, nabumetone, and meloxicam. However, aspirin is not included as a drug in the traditional NSAIDs. This is because aspirin has a unique mechanism of action and are usually used on cardiovascular patients. Specific COX-2 inhibitors (COXIBs) are defined as drugs with any of the following compound: celeCoxib, rofeCoxib, valdeCoxib, pareCoxib and etoriCoxib.

### Covariates

In order to be as comprehensive as possible in adjusting for factors that might affect the outcome, we looked at published risk factors for TB and identified 71 covariates (Table 1) [1, 19, 20]. All covariates were assessed before NSAID exposure, except for the one-year period used for exposure assessment. Using ICD9-CM codes, the following covariates were assessed: age, sex, burden of comorbid conditions, indicators for frailty, risk factors for TB disease and use of specific medications. Risk factors for TB included alcoholism-related disease (291, 303, 305.0, 357, 425, 535.3, 571.0-571.3, 577.1), diabetes mellitus (DM) (250), chronic renal failure or hemodialysis (585, V56.0), solid organ transplantation (V42), COPD (491,492, 496), silicosis (502), presence of cancer (140-239), malnutrition-related disorders such as cachexia, anorexia, abnormal loss of weight (799.4, 263, 783.2, 783.3), and postgastric surgery (564.2). Use of specific medications included aspirin (N02BA), systemic immunosuppressive agents and biologics (L04A), systemic corticosteroids, and disease modifying anti-rheumatic drugs (M01B, M01C). Each individual’s burden of comorbidity was quantified by a combined weighted comorbidity index. This index is an improved Charlson Index that combines the Elixhauser system [21]. The score contains common comorbidities such as myocardial infarction, congestive heart failure, peripheral vascular disease, cerebrovascular disease, dementia, chronic pulmonary disease, connective tissue disease, ulcer disease, liver disease, diabetes, hemiplegia, renal disease, neoplasms and AIDS.

**Table 1:** Participant enrollment and baseline characteristics

	Total number of control= 470600 person-years			
	Traditional NSAIDs (*N*=123,419 person years)	Coxibs (*N*=16,392 person years)	Unexposed (*N*=340,396 person years)	*P*-value
Number of TB cases	1407	206	3206	N.A
Demographics				
Male sex (%)	84623 (68.6)	10420 (63.6)	245077 (72.0)	<.0001
Age mean (year)	63.1±17.5	75.5±9.8	55.9±19.9	<.0001
Index year (%)				
1999	8740 (7.1)	0	24660 (7.2)	<.0001
2000	10496 (8.5)	0	28004 (8.2)	
2001	10168 (8.2)	80 (0.5)	26409 (7.8)	
2002	11097 (8.9)	990 (6.0)	29178 (8.6)	
2003	11271 (9.1)	2104 (12.8)	29385 (8.6)	
2004	10658 (8.6)	2463 (15.0)	28264 (8.3)	
2005	11368 (9.2)	2122 (13.0)	30022 (8.8)	
2006	9328 (7.6)	1143 (6.9)	26013 (7.6)	
2007	8046 (6.5)	1091 (6.7)	22810 (6.7)	
2008	9164 (7.4)	1429 (8.7)	26625 (8.1)	
2009	7438 (6.0)	1452 (8.9)	22080 (6.5)	
2010	7942 (6.4)	1704 (10.4)	23837 (7.0)	
2011	7585 (6.2)	1814 (11.1)	21827 (6.4)	
Area : urban region	52030 (42.2)	6788 (41.4)	169458 (49.8)	<.0001
Area : metro area	31446 (25.5)	3897 (23.8)	85565 (25.1)	
Area : suburban area	26117 (21.2)	3531 (21.5)	59604 (17.5)	
Area : countryside area	13826 (11.2)	2176 (13.3)	25769 (7.6)	
Annual insurance premium				
Dependent	10446 (8.6)	2349 (14.6)	28046 (8.3)	<.0001
<666 USD	37517 (30.7)	6355 (39.5)	90984 (26.9)	
666-1331 USD	52657 (43.2)	5914 (36.8)	139440 (41.3)	
>= 1331 USD	21424 (17.6)	1462 (9.1)	79315 (23.5)	
Comorbidity score				
Baseline combined comorbidity score	1.41±1.94	2.54±2.43	0.84±1.54	<.0001
Individual comorbidity				
Peripheral vascular disease	9704 (7.9)	2539 (15.8)	13440 (4.0)	<.0001
Congestive heart failure	14689 (12.0)	4049 (25.2)	20203 (6.0)	<.0001
Myocardial infarction/acute coronary syndromes	3842 (3.2)	947 (5.9)	6008 (1.8)	<.0001
Cerebrovascular disease	25397 (20.8)	6550 (40.7)	40531 (12.0)	<.0001
Dementia	4412 (3.6)	1439 (8.9)	9242 (2.7)	<.0001
Chronic pulmonary disease	51073 (41.9)	10138 (63.1)	86738 (25.7)	<.0001
Rheumatologic disease	4587 (3.8)	1231 (7.7)	5615 (1.7)	<.0001
Peptic ulcer disease	51084 (41.9)	10386 (64.6)	84019 (24.9)	<.0001
Mild liver disease	39309 (32.2)	6968 (43.3)	68469 (20.3)	<.0001
Diabetes without chronic complications	26942 (22.1)	5782 (35.9)	45868 (13.6)	<.0001
Diabetes with chronic complications	8356 (6.9)	2053 (12.8)	13954 (4.1)	<.0001
Hemiplegia or paraplegia	5199 (4.3)	1263 (7.9)	9365 (2.8)	<.0001
Renal disease	11377 (9.3)	2978 (18.5)	16967 (5.0)	<.0001
Any malignancy, including leukemia and lymphoma	11991 (9.8)	2900 (18.0)	19535 (5.8)	<.0001
Moderate or severe liver disease	690 (0.6)	157 (1.0)	1252 (0.4)	<.0001
Metastatic solid tumor	1245 (1.0)	271 (1.7)	2041 (0.6)	<.0001
AIDS/HIV	84 (0.07)	15 (0.09)	154 (0.05)	0.003
Alcohol/drug use	3425 (2.8)	549 (3.4)	6826	<.0001
Psychiatric disorder	43667 (35.8)	8652 (53.8)	66012 (19.5)	<.0001
Neurologic disorder	7046 (5.8)	2069 (12.9)	12232 (3.6)	<.0001
Obesity	1194 (0.9)	223 (1.4)	1892 (0.6)	<.0001
Other Cancer except Metastatic solid tumor	36548 (29.9)	7072 (43.9)	68592 (20.3)	<.0001
COPD	37556 (30.8)	8128 (50.6)	58896 (17.4)	<.0001
Silicosis	218 (0.2)	32 (0.2)	258 (0.1)	<.0001
Gastrointestinal or esophageal hemorrhage	7319 (6.0)	1865 (11.6)	12848 (3.8)	<.0001
Risk factors				
Pregnancy	913 (0.8)	49 (0.3)	3171 (0.9)	<.0001
bed-ridden status	1561 (1.3)	361 (2.3)	2408 (0.7)	<.0001
Solid organ transplantation such as renal or heart transplantation	15 (0.01)	2 (0.01)	77 (0.02)	0.04
Malnutrition	678 (0.6)	129 (0.8)	928 (0.3)	<.0001
Postgastric surgery	34 (0.03)	7 (0.04)	30 (0.01)	<.0001
Healthcare service utilization in the previous year				
The number of outpatient department visits	32.4±25.1	42.1±27.6	14.1±15.9	<.0001
The number of emergency department visits	0.2±0.87	0.4±1.26	0.1±0.51	<.0001
The number of hospitalizations	0.3±0.87	0.5±1.12	0.2±0.9	<.0001
Medication use				
Aspirin	25603 (20.9)	5118 (31.8)	32912 (9.7)	<.0001
Systemic immunosuppressive agents and biologics	416 (0.3)	284 (1.8)	336 (0.1)	<.0001
Systemic corticosteroids	26268 (21.5)	4526 (28.2)	23802 (7.1)	<.0001
DMARDs (disease modifying anti-rheumatic drugs)	2165 (1.8)	694 (4.3)	2150 (0.6)	<.0001
Statin	9380 (7.7)	2233 (13.9)	14224 (4.2)	<.0001
ACE inhibitors	17324 (14.2)	2925 (18.2)	23278 (6.9)	<.0001
Beta blockers	21155 (17.3)	3771 (23.5)	27789 (8.2)	<.0001
Loop diuretics	8569 (7.0)	2295 (14.3)	9196 (2.7)	<.0001
Angiotensin II antagonists	12596 (10.3)	3789 (23.6)	19482 (5.8)	<.0001
Digoxin	2925 (2.4)	690 (4.3)	4265 (1.3)	<.0001
Nitrates	9178 (7.5)	2133 (13.3)	11539 (3.4)	<.0001
Antipsychotics	434 (0.4)	129 (0.8)	1190 (0.4)	0.01
Proton-pump inhibitors(PPI)	5977 (4.9)	1606 (9.9)	8098 (2.4)	<.0001
CA channel blocker	33842 (27.7)	7126 (44.3)	47053 (13.9)	<.0001
Acetaminophen	63673 (52.2)	8965 (55.8)	78967 (23.4)	<.0001

### Data analysis

We estimated the rate ratio by using a time matched case control-sampling scheme [22]. Incidence rate ratios of active TB (plus 95% confidence intervals [CIs]) were estimated by conditional logistic regression analysis adjusted for all covariates. Disease risk score (DRS) was used to adjust for confounders, and compared the different exposures in a case-control study design [23]. A study-specific DRS was constructed to balance disease risks between different drug exposure groups. On Additional file 1, we reported the c- statistic (0.81) of the DRS model, component variables, and the respective weights of the component variables. The DRS was defined as the probability of developing active TB among the participants unexposed to NSAID or Coxibs based on the individual’s baseline covariates. To estimate DRS, we carried out multivariate logistic regression analysis, where active TB was treated as the dependent variable, and all empirical clinical predictors were treated as independent variables. Hence, the DRS characterize the relationship between potential risk factors and active TB. To avoid the potential unrealistic linear assumption of continuous variables in the regression model, we carried out a fractional polynomial DRS (fpDRS) adjustment.

We also carried out a dose response analyses and subgroup analyses in high-risk patients, to further assess the robustness of our results. Predefined subgroups included sex and age of 70 years. Attributable risk percentage and population attributable risk percentage were also calculated (Additional file 2). All analyses were carried out with SAS 9.3 for Windows (SAS Institute Inc, Cary, NC) and the data are reported in accordance with STROBE guidance.

## Results

### Participant enrollment and baseline characteristics

Table 1 shows the baseline characteristics of the cohort. The source population comprises of 1,000,000 participants. After exclusion of prevalent users of NSAIDs and prevalent cases of TB in the 1 year pre-enrollment period, 123,419 person years were exposed to traditional NSAIDs and 16,392 person years were exposed to Coxibs. Coxibs users were found to increase steadily from 2001 to 2004. However, there is a sharp decline in Coxib users after 2004, when rofecoxib revealed safety concerns and was pulled off the market. The use of Coxib was found to increase steadily again from 2007 to 2011. There were significant differences in the age, and Charlson index comorbidity score of the different participants. In general, Coxibs users were the oldest (75.5 ± 9.8 years old) and had the highest comorbidity score (2.54 ± 2.43), while the unexposed control was the youngest (55.9 ± 19.9 years old) and had the lowest comorbidity score (0.84 ± 1.54). Traditional NSAIDs users had an average age and comorbidity score in between that of Coxibs users and unexposed control. The number of comorbidities, known risk factors and medications also tend to agree with the pattern observed in the comorbidity score. Coxibs users generally had a higher number of comorbidities, known risk factors and medications usage, then followed by traditional NSAIDs users and finally unexposed users.

### Use of NSAIDs and risk of active TB onset

We identified 340,396 number of participants unexposed to traditional NSAIDs and Coxibs in our cohort, of which 3,206 developed active TB. The crude TB incidence rate for the unexposed control is 0.93% (3,206/343,602 person-years). (Table 1) In our cohort, 1,407 traditional NSAIDs users were found to be associated with active TB, resulting in a crude incidence rate of 1.13% (1,407/124,826 person-years). We also identified 206 Coxibs users associated with active TB, resulting in a crude incidence rate of 1.24% (206/16,598 person-years).

Table 2 shows the rate ratios associating the use of NSAIDs within 31-90 days, on the risk of new active TB onset. The first method, which is matching only on agegroup, gender, and calendar year, can be considered an unadjusted way of obtaining rate ratio (RR). The age-sex-year matched RR is 1.39 (95%CI, 1.24 – 1.57) for traditional NSAIDs and 1.40 (95%CI, 1.03 – 1.92) for Coxibs. Adjustment for individual confounders attenuated the RR to 1.31 (95%CI, 1.15 – 1.49) for use of traditional NSAIDs and 1.18 (95%CI, 0.85 – 1.63) for use of Coxibs. To balance the disease risk in multiple drug exposure groups independent of the changing indications for NSAIDs, a disease risk score (DRS) was created. The effect estimates after DRS adjustment or fractional polynomial (fp) DRS adjustment was similar to the adjustment by individual confounders. In all the different type of adjustment, use of traditional NSAIDs was associated with a significantly increased risk for TB, but the use of Coxibs was not associated with a significantly increased risk for TB.

**Table 2:** Crude and adjusted effect measure for the association between use of NSAIDs and risk of active TB

	Effect estimate matched on age group, gender, and year (RR, 95% confidence interval)	Confounder adjusted effect estimate (RR, 95% confidence interval)	Disease Risk Score adjusted effect estimate (RR, 95% confidence interval)	Fractional Polynomial Disease Risk Score adjusted effect estimate (RR, 95% confidence interval)
Use of Traditional NSAIDs	1.39 (1.24 – 1.57)^a^	1.31 (1.15 –1.49)^a^	1.30 (1.15– 1.47)^a^	1.19 (1.05 1.35)^a^
Use of Coxibs	1.40 (1.03 – 1.92)^a^	1.18 (0.85 – 1.63)	1.23 (0.89 – 1.70)	1.07 (0.78 – 1.48)

^a^Refers to result that is statistically significant

*RR* refers to rate ratio

### Duration-response analysis

Table 3 investigates whether the use of traditional NSAIDs with a different duration can affect the risk of active TB. The cumulative incidence of active TB for the non NSAID user (5.74%), is lower than either the 7-14 days of NSAID user (8.95%) or >14 days of NSAID user (9.00%). After fpDRS adjustment of multiple covariates, use of 7-14 days of NSAID ([ARR], 1.46; 95%CI, 1.34 – 1.59) or >14 days’ use of traditional NSIADs ([ARR], 1.25; 95%CI, 1.04 – 1.49) was still associated with higher risk than the reference. However, no duration response effect was observed.

**Table 3:** Relationship between number of days that participants are prescribed with traditional NSAIDs and risk of active TB

	Risk estimate by different duration category/drug use	
Total number of days using traditional NSAIDs	Cumulative Incidence (case/person)	Fractional Polynomial Disease Risk Score adjusted RR (95% CI)
0-7days (reference)	5.74%	NA
7-14 days	8.95%	1.46 (1.34 – 1.59)^a^
>14 days	9.00%	1.25 (1.04 – 1.49)^a^

^a^Refers to result that is statistically significant

*RR* refers to rate ratio

## Discussion

In this population-based study based on one million national representative participants, we found that use of traditional NSAIDs was at increased risk of active TB. No duration response effect was observed in the 90-day risk period.

NSAIDs are one of the most widely used medications in the world. According to a French national healthcare insurance system study from 2009-2010, about 43.6% of participants have used some form of NSAID at least once [5]. Thus, it is vital for us to report the contribution of NSAIDs to TB burden. In our cohort, it was found that new user of traditional NSAIDs accounts for approximately 26% cases of active TB among patients taking these medications (Additional file 2). By defining greater than 7 days’ prescription of NSAID as a user, we found that 9.4% of Taiwanese had used traditional NSAIDs and 2.1% had used Coxibs in the year 2011. Using the above percentages, we can conclude that use of traditional NSAID accounts for 3.2% cases of active TB among the entire Taiwan population (see Additional file 2 for the attributable risk/ population attributable risk fraction calculation). Therefore, the risk of TB associating with the use of traditional NSAIDs is quite high and cannot be ignored by the health policy makers in Taiwan.

In general, our study agreed with three clinical reports associating the use of NSAIDs with an increase in TB risk [12–14]. However, our crude and adjusted estimates were often found lower than the Chang’s group, but in line with the Brassard group [11, 14]. Part of the disparity could be due to the significant differences in our study design. The Chang’s study cohort consists of patients with psoriasis or psoriatic arthritis but we were looking at the general population. As a result, there would be significant differences in the baseline characteristics of participants. In fact, we observed that 39.2% of Chang’s control is prescribed with corticosteroids as compared to 7.1% of our control. It is unknown how many NSAID users in Chang’s study were prescribed with corticosteroids, but it is likely to be higher than ours. Immunosuppressive drugs such as corticosteroids have long been associated with the risk of tuberculosis and can definitely contribute to the higher risk observed in Chang’s study [1, 14].

Results of our study should be interpreted in light of both strength and weakness. To the best of our knowledge, this is the biggest TB and NSAIDs study that has ever been carried out. In addition, we constructed a database specific DRS by comprehensively including a large number of covariates (71) and allowed a nonlinear fractional polynomial term to enter the model, which we believed may greatly relieve the concerns of residual confounding in a claim database research. We chose not to construct a propensity score because the indications for Coxib use have changed greatly in the past 10 years. The DRS can balance the disease risk among multiple drug exposure groups independent of the changing indications for NSAIDs, and can adjust for several rare covariates in the subgroup analysis [23]. Despite the comprehensiveness of the DRS and a large number of covariates, we recognized that unmeasured confounding could still be an issue. Since we were using a claims database for our study, many lifestyle factors such as smoking, alcohol, and body mass index are missing. Smoking and excessive alcohol consumption can increase TB risk by 2-3 fold [1]. We attempted to adjust for these missing confounder by including related diseases such as chronic obstructive pulmonary disease, liver disease, coronary artery disease, malnutrition and alcohol related disorders.

In addition, the unknown number of over the counter use of NSAIDs in the control group might also affect our result. However, we believed that there are few incentives for ≧ 7 days use of over the counter NSAIDs. In Taiwan, the purchase of 7 days of over the counter Ibuprofen actually costs more than the reimbursed cost of medication and the physician visit. Thus, there is little incentive for patients to purchase over the counter traditional NSAIDs. However, if we were to account for the over the counter use of NSAID, the result will be likely biased towards the null due to nondifferential misclassification.

Another limitation inherent in our database study was that active TB disease was defined on the basis of ICD-9 codes with compatible anti-TB prescription history. Since microbiological data is lacking, there is potential for misclassification of TB users. However, we believed that there was little misclassification of TB users, as past linked survey data showed that the definition is highly accurate [19, 20].

Protopathic bias, interpreted as fever symptoms preceding active TB disease leading to the prescription of NSAIDs, is another possible cause for the observed association [24]. However, we try to limit protopathic bias for users of NSAIDs in several ways. First, we looked at long-term users of NSAIDs (>7 days) and exclude short-term NSAID users, who have a higher probability of protopathic bias. By restricting to traditional NSAID users having a drug prescription record ≧ 7 days, we found that most of the patients are associated with codes for arthritis and other rheumatic conditions, and there are relatively fewer patients associated with codes for fever. Second, use of NSAID was defined as having a drug prescription record ≧ 7 days that was filled between 31 and 90 days prior to the TB diagnosis. There is evidence showing that the mean delay between the first medical visits and the confirmed diagnosis of TB is less than 30 days. The mean intervals between patients’ first medical visit to the definite diagnosis were reported to be < 1 day, 6.07 days and 25.53 days in outpatient clinics, the emergency department and hospitalization, respectively [25]. Thus, taking the study design and the medical landscape in Taiwan together, we think that protopathic bias could play a part, but could not contribute entirely to the increase in TB risk associated with NSAIDs.

## Conclusion

In conclusion, using a large population-based database we have identified an association between traditional NSAID use and active TB. We did not find evidence for a causative mechanism between traditional NSAIDs and TB, and more research is required to verify whether the association between traditional NSAIDs and TB is causal, or simply reflects an increased use of anti-inflammatory drugs in the early phases of TB onset.

## Additional file

Additional file 1: Empirical predictors of incident active TB and associated rate ratios of the disease risk score model. (DOC 92 kb)
Additional file 2: Calculation of attributable risk fraction and population attributable risk fraction of TB. (DOC 63 kb)

## Abbreviation

CI: Confidence interval
COX-1: Cyclooxygenase-1
COX-2: Cyclooxygenase-2
Coxibs: COX-2 inhibitors
NSAIDs: Non-steroidal anti-inflammatory drugs
TB: *Mycobacterium tuberculosis*

## References

[1] Lawn SD, Zumla AI (2011). Tuberculosis. Lancet.

[2] Tuberculosis (TB) [http://www.who.int/gho/tb/en/index.html].

[3] Paulose-Ram R, Hirsch R, Dillon C, Losonczy K, Cooper M, Ostchega Y (2003). Prescription and non-prescription analgesic use among the US adult population: results from the third National Health and Nutrition Examination Survey (NHANES III). Pharmacoepidemiol Drug Saf.

[4] Tielemans MM, Eikendal T, Jansen JB, van Oijen MG (2010). Identification of NSAID users at risk for gastrointestinal complications: a systematic review of current guidelines and consensus agreements. Drug Saf.

[5] Duong M, Salvo F, Pariente A, Abouelfath A, Lassalle R, Droz C, Blin P, Moore N (2014). Usage patterns of ‘over-the-counter’vs. prescription-strength nonsteroidal anti-inflammatory drugs in France. Br J Clin Pharmacol.

[6] Flower R, Gryglewski R, Herbaczynska-Cedro K, Vane JR (1972). Effects of anti-inflammatory drugs on prostaglandin biosynthesis. Nat New Biol.

[7] Ferreira SH, Moncada S, Vane JR (1973). Prostaglandins and the mechanism of analgesia produced by aspirin-like drugs. Br J Pharmacol.

[8] Vane JR (1971). Inhibition of prostaglandin synthesis as a mechanism of action for aspirin-like drugs. Nature.

[9] Bombardier C, Laine L, Reicin A, Shapiro D, Burgos-Vargas R, Davis B, Day R, Ferraz MB, Hawkey CJ, Hochberg MC, VIGOR Study Group (2000). Comparison of upper gastrointestinal toxicity of rofecoxib and naproxen in patients with rheumatoid arthritis. N Engl J Med.

[10] Silverstein FE, Faich G, Goldstein JL, Simon LS, Pincus T, Whelton A, Makuch R, Eisen G, Agrawal NM, Stenson WF (2000). Gastrointestinal toxicity with celecoxib vs nonsteroidal anti-inflammatory drugs for osteoarthritis and rheumatoid arthritis: the CLASS study: A randomized controlled trial Celecoxib Long-term Arthritis Safety Study. JAMA.

[11] Chang CH, Chen HC, Lin JW, Kuo CW, Shau WY, Lai MS (2011). Risk of hospitalization for upper gastrointestinal adverse events associated with nonsteroidal anti-inflammatory drugs: a nationwide case-crossover study in Taiwan. Pharmacoepidemiol Drug Saf.

[12] Chen YJ, Wu CY, Shen JL, Chen TT, Chang YT (2013). Association between traditional systemic antipsoriatic drugs and tuberculosis risk in patients with psoriasis with or without psoriatic arthritis: results of a nationwide cohort study from Taiwan. J Am Acad Dermatol.

[13] Tomasson H, Brennan M, Bass M (1984). Tuberculosis and nonsteroidal anti-inflammatory drugs. Can Med Assoc J.

[14] Brassard P, Lowe AM, Bernatsky S, Kezouh A, Suissa S (2009). Rheumatoid arthritis, its treatments, and the risk of tuberculosis in Quebec, Canada. Arthritis Rheum.

[15] Lu J-FR, Hsiao WC (2003). Does universal health insurance make health care unaffordable? Lessons from Taiwan. Health Affairs.

[16] Lee MS, Lin RY, Chang YT, Lai MS (2012). The risk of developing non-melanoma skin cancer, lymphoma and melanoma in patients with psoriasis in Taiwan: a 10-year, population-based cohort study. Int J Dermatol.

[17] Chang CH, Lin JW, Wu LC, Lai MS, Chuang LM, Chan KA (2012). Association of thiazolidinediones with liver cancer and colorectal cancer in type 2 diabetes mellitus. Hepatology.

[18] Ray WA (2003). Evaluating medication effects outside of clinical trials: new-user designs. Am J Epidemiol.

[19] Baker MA, Lin HH, Chang HY, Murray MB (2012). The risk of tuberculosis disease among persons with diabetes mellitus: a prospective cohort study. Clin Infect Dis.

[20] Lin HH, Ezzati M, Chang HY, Murray M (2009). Association between tobacco smoking and active tuberculosis in Taiwan: prospective cohort study. Am J Respir Crit Care Med.

[21] Gagne JJ, Glynn RJ, Avorn J, Levin R, Schneeweiss S (2011). A combined comorbidity score predicted mortality in elderly patients better than existing scores. J Clin Epidemiol.

[22] Lubin JH, Gail MH (1984). Biased selection of controls for case-control analyses of cohort studies. Biometrics.

[23] Glynn RJ, Gagne JJ, Schneeweiss S (2012). Role of disease risk scores in comparative effectiveness research with emerging therapies. Pharmacoepidemiol Drug Saf.

[24] Horwitz RI, Feinstein AR (1980). The problem of “protopathic bias” in case-control studies. Am J Med.

[25] Wu Y-C, Hsu G-J, Chuang KY-C, Lin R-S (2007). Intervals before tuberculosis diagnosis and isolation at a regional hospital in Taiwan. J Formos Med Assoc.

