# Supplementary material for: Risk of incident active tuberculosis disease in patients treated with non-steroidal anti-inflammatory drugs: a population-based study
Source: BMC Pulm Med. 2017 May 4;17:82. doi: 10.1186/s12890-017-0425-3 (PMC5418697; doi:10.1186/s12890-017-0425-3)
Supplement: Additional file 1: — Empirical predictors of incident active TB and associated rate ratios of the disease risk score model. (DOC 92 kb) [file 12890_2017_425_MOESM1_ESM.doc]

**Additional file 1. Empirical predictors of incident active TB and associated rate ratios of the disease risk score model**

| **Characteristics** | **IRR (95%CI) for DRS** | **P-value** |
| --- | --- | --- |
| **AUC** | **0.81** |  |
| **Demographics** | | |
| Gender : F | 0.45 (0.43 – 0.47) | <.0001 |
| Age | 1.02 (1.01 – 1.03) | <.0001 |
| Index year : reference 2011 |  |  |
| 1999 | 2.13 (1.87 – 2.43) | <.0001 |
| 2000 | 2.02 (1.78 – 2.29) | <.0001 |
| 2001 | 1.87 (1.65 – 2.12) | <.0001 |
| 2002 | 1.94 (1.71 – 2.19) | <.0001 |
| 2003 | 1.83 (1.62 – 2.07) | <.0001 |
| 2004 | 1.89 (1.68 – 2.14) | <.0001 |
| 2005 | 1.84 (1.64 – 2.08) | <.0001 |
| 2006 | 1.52 (1.34 – 1.72) | <.0001 |
| 2007 | 1.35 (1.19 – 1.53) | <.0001 |
| 2008 | 1.38 (1.22 – 1.56) | <.0001 |
| 2009 | 1.22 (1.08 – 1.38) | 0.0002 |
| 2010 | 1.14 (1.01 – 1.30) | 0.04 |
| **Living area : reference countryside area** |  |  |
| Area : urban region | 0.78 (0.69 – 0.88) | <.0001 |
| Area : metro area | 0.82 (0.72 – 0.95) | <.0001 |
| Area : suburban area | 0.87 (0.76 – 0.99) | 0.01 |
| **Annual insurance premiums : reference dependent** |  |  |
| <666 USD | 1.09 (1.00 – 1.19) | 0.04 |
| 666-1331 USD | 0.92 (0.84 – 1.00) | 0.06 |
| >= 1331 USD | 0.58 (0.53 – 0.65) | <.0001 |
| **Comorbidity score** | | |
| Baseline combined comorbidity score | 1.24 (1.20 – 1.29) | <.0001 |
| Baseline combined comorbidity score : quadratic | 0.98 (0.97 – 0.99) | <.0001 |
| **Individual comorbidity** | | |
| Peripheral vascular disease | 0.79 (0.69 – 0.91) | 0.0007 |
| Congestive heart failure | 0.85 (0.77 – 0.94) | 0.001 |
| Myocardial infarction/acute coronary syndromes | 1.09 (0.95 – 1.25) | 0.21 |
| Cerebrovascular disease | 0.95 (0.88 – 1.03) | 0.24 |
| Dementia | 0.97 (0.86 – 1.09) | 0.58 |
| Chronic pulmonary disease | 1.20 (1.11 – 1.30) | <.0001 |
| Rheumatologic disease | 1.10 (0.97 – 1.25) | 0.15 |
| Peptic ulcer disease | 0.98 (0.92 – 1.03) | 0.37 |
| Mild liver disease | 0.92 (0.87 – 0.97) | 0.004 |
| Diabetes without chronic complications | 1.18 (1.11 – 1.26) | <.0001 |
| Diabetes with chronic complications | 1.24 (1.13 – 1.36) | <.0001 |
| Hemiplegia or paraplegia | 0.96 (0.85 – 1.10) | 0.57 |
| Renal disease | 1.08 (0.99 – 1.18) | 0.06 |
| Any malignancy, including leukemia and lymphoma | 1.02 (0.93 – 1.12) | 0.70 |
| Moderate or severe liver disease | 1.08 (0.86 – 1.35) | 0.50 |
| Metastatic solid tumor | 0.79 (0.62 – 1.00) | 0.05 |
| AIDS/HIV | 2.92 (1.72 – 4.96) | <.0001 |
| Alcohol/drug abuse | 1.68 (1.51 – 1.87) | <.0001 |
| Psychiatric disorder | 0.88 (0.83 – 0.93) | <.0001 |
| Neurologic disorder | 1.17 (1.06 – 1.28) | <.0001 |
| Obesity | 0.42 (0.28 – 0.61) | <.0001 |
| Other Cancer except Metastatic solid tumor | 0.94 (0.88 – 1.00) | 0.06 |
| COPD | 1.25 (1.16 – 1.36) | <.0001 |
| Silicosis | 1.29 (0.77 – 2.15) | 0.33 |
| Gastrointestinal or esophageal hemorrhage | 1.21 (1.11 – 1.32) | <.0001 |
| **Risk factors** | | |
| Pregnancy | 1.15 (0.92 – 1.45) | 0.21 |
| bed-ridden status | 0.95 (0.77 – 1.17) | 0.62 |
| Solid organ transplantation such as renal or heart transplantation | 1.62 (0.72 – 3.65) | 0.23 |
| Malnutrition | 1.34 (1.09 – 1.65) | 0.008 |
| Postgastric surgery | 2.09 (0.86 – 5.09) | 0.10 |
| **Healthcare service utilization** | | |
| The number of OPD visit | 1.01 (1.01 – 1.02) | <.0001 |
| The number of OPD visit : quadratic | 1.00 (1.00 – 1.00) | <.0001 |
| The number of emergency department visit | 1.05 (1.01 – 1.10) | 0.02 |
| The number of emergency department visit : quadratic | 0.99 (0.99 – 1.10) | 0.05 |
| The number of hospitalization | 1.33 (1.27 – 1.39) | <.0001 |
| The number of hospitalization : quadratic | 0.98 (0.98 – 0.99) | <.0001 |
| **Medication use** | | |
| Aspirin | 0.97 (0.90 – 1.04) | 0.40 |
| Systemic immunosuppressive agents and biologics | 1.39 (0.99 – 1.96) | 0.06 |
| Systemic corticosteroids | 1.27 (1.19 – 1.35) | <.0001 |
| DMARDs (disease modifying anti-rheumatic drugs) | 1.35 (1.13 – 1.62) | <.0001 |
| Statin | 0.76 (0.68 – 0.85) | <.0001 |
| ACE inhibitors | 0.93 (0.86 – 1.00) | 0.08 |
| Beta blockers | 0.88 (0.82 – 0.95) | 0.0007 |
| Loop diuretics | 1.02 (0.93 – 1.12) | 0.69 |
| Angiotensin II antagonists | 0.84 (0.77 – 0.93) | 0.0003 |
| Digoxin | 1.04 (0.90 – 1.20) | 0.62 |
| Nitrates | 0.93 (0.83 – 1.03) | 0.16 |
| Antipsychotics | 0.78 (0.59 – 1.05) | 0.10 |
| Proton-pump inhibitors(PPI) | 0.96 (0.86 – 1.07) | 0.50 |
| CA channel blocker | 0.89 (0.83 – 0.95) | 0.0007 |
| Acetaminophen | 1.11 (1.05 – 1.17) | <.0001 |
