# Supplementary material for: Risk of incident active tuberculosis disease in patients treated with non-steroidal anti-inflammatory drugs: a population-based study
Source: BMC Pulm Med. 2017 May 4;17:82. doi: 10.1186/s12890-017-0425-3 (PMC5418697; doi:10.1186/s12890-017-0425-3)
Supplement: Additional file 2: — Calculation of attributable risk fraction and population attributable risk fraction of TB. (DOC 63 kb) [file 12890_2017_425_MOESM2_ESM.doc]

# Additional file 2: Calculation of attributable risk fraction and population attributable risk fraction of TB

**Attributable risk fraction**

$$\frac{\left(RR-1\right)}{RR}\times100\%$$

where RR = relative risk

Traditional Nsaids:

0.35/1.35 = 26%

**Population attributable risk fraction**

$$\begin{matrix} \left\{\frac{P(E)\times\left(RR-1)\right)}{P(E)\times\left(RR-1\right)+1}\right\}\times100\% \\ \end{matrix}$$

where P(E) = prevalence of exposure (i.e. 9.4% for traditional NSAID and 2.1% for Coxib in year 2011)

Traditional Nsaids :

0.094*0.35/(0.094*0.35+1) = 3.2%
